# Age Structure, Body Size, and Sexual Dimorphism in a High-Altitude Population of *Pelophylax ridibundus* (Pallas, 1771)

**DOI:** 10.3390/ani14223230

**Published:** 2024-11-11

**Authors:** Serkan Gül, Cantekin Dursun, Ceren Tabak, Sümeyye Büyüksofuoğlu, Nurhayat Özdemir

**Affiliations:** 1 Department of Biology, Faculty of Arts and Sciences, Recep Tayyip Erdoğan University, 53100 Rize, Türkiye; serkan.gul@erdogan.edu.tr (S.G.); ceren_tabak21@erdogan.edu.tr (C.T.); sumeyye_buyuksofuoglu20@erdogan.edu.tr (S.B.); 2 Department of Biology, Faculty of Science, Karadeniz Technical University, 61080 Trabzon, Türkiye; nurhayat@ktu.edu.tr

**Keywords:** marsh frog, von Bertalanffy, weight, shape, life history, Anatolia

## Abstract

This study aimed to describe the morphological characteristics and age structure of a *P. ridibundus* population sampled from a high altitude. For this purpose, a total of 54 adult frogs (33 males and 21 females) were collected from Şavşat district, Artvin province, Türkiye. The samples were aged based on skeletochronology method. Furthermore, von Bertalanffy growth curve models were constructed using SVL and weight data. To assess sexual size and shape dimorphism, the measurements of 23 different morphological characters were analyzed using univariate and multivariate statistical techniques. The mean SVL and weight were found to be 78.05 mm and 60.87 g for males and 93.95 mm and 109.61 g for females. The mean age was 3.67 years and 4.05 years for males and females, respectively. The maximum age was found to be 6 years for both sexes. Growth curve models revealed a significant relationship between age, SVL, and weight. A clear female-biased sexual size dimorphism was found in all morphological characters. However, SVL-adjusted values indicated that males had larger head and limb structures resulting from evolutional and sexual pressures associated with reproduction and male–male competition. The shape dimorphism was found only in forelimbs, and it was male-biased and was related to clasping in amplexus.

## Simple Summary

The age structure of amphibian populations plays an essential role in understanding population dynamics, potential impacts of environmental change, and conservation status. The Marsh frog *Pelopyhlax ridibundus* has become a subject of various studies to investigate age structure and body size variation. However, the populations living at high altitudes were paid less attention to while describing life history traits. Here, sexual dimorphic traits, life history characteristics, and relationship between age and body size/weight are presented for a population located at an altitude of over 2000 m. Accordingly, female samples were larger in terms of body size and body weight. However, male-biased dimorphism was observed in the head and forelimbs and was caused by adaptation to reproduction success. Females had greater mean age than males, but the maximum age (i.e., 6 years) was found identical in both sexes.

## 1. Introduction

Amphibians are an ecologically and evolutionarily important group of animals capable of living in terrestrial and aquatic habitats [1]. The age structure of amphibian populations plays an essential role for understanding population dynamics, potential impact of environmental changes, and conservation status. This structure representing the distribution of individuals in different age groups can serve as an indicator while assessing demographic characteristics of amphibians in terms of population health and sustainability [2]. In addition, the age at which amphibians reach sexual maturity plays a crucial role in their protection. Delayed maturation, reproductive challenges, habitat destruction, disease, and population decline due to climate change significantly reduce their ability to avoid extinction [3]. Additionally, the relationship between age structure and body size in amphibians is an important area of research that supplies insights into amphibian growth patterns, population dynamics, and survival strategies. This relationship is shaped by the effect of biological and environmental components, including life history strategies of the species, habitat conditions, and environmental pressures such as disease and predation [4,5,6]. Moreover, altitude, an important environmental factor, has significant impacts on both age structure and body size of amphibians. The physiological and ecological changes regarding altitude, such as temperature, oxygen availability, and habitat characteristics, affect amphibian development, growth, and survival in complex ways [7,8,9].

Skeletochronology is a widely used method for determining the age structure of amphibians as well as life history traits and population dynamics. In this method, the age calculation is performed using a cross-section of diaphysis from phalanges and counting the lines of arrested growth (LAGs) regarding seasonal cycles of growth and stagnancy. Furthermore, skeletochronology is essential to determine the lifespan of the amphibian population and to describe sexual dimorphism and the interaction between these parameters [10]. The reliability of skeletochronology is demonstrated with methodological studies comparing the lectures of different bones of the same individual or studies that compare the age estimates from mark recapture and skeletochronology [11,12].

*Pelophylax ridibundus* (Pallas, 1771), commonly known as the marsh frog, is distributed in most of Europe, Balkans, Uzbekistan, Western Asia including whole Anatolia, Western Kazakhstan, and Siberia [13,14]. This species usually prefers to live in stagnant and slow-flowing freshwater bodies and has a wide ecological tolerance [15]. Various studies have been conducted on the age structure of different populations of this species [16,17,18,19,20,21]. However, there is no study on a population above 2000 m altitude in Türkiye. This study aimed (1) to find the effect of altitude on age structure, body size, and life history traits of *P. ridibundus* population, (2) to compare differences in age structure, body size, and body weight between sexes, and (3) to expand knowledge about the ecology of the species thanks to these insights.

## 2. Materials and Methods

### 2.1. Sampling and Laboratory Process

**Figure 1:**
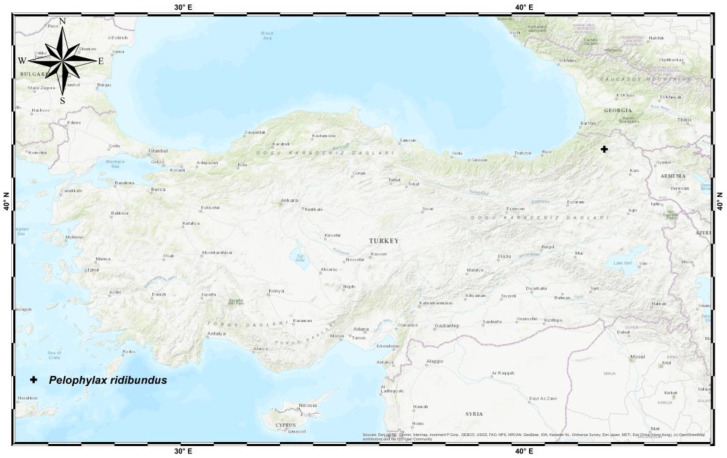
The geographic demonstration of the study area (41.166 N; 42.394 E).

A total of 54 adult frogs (33 males and 21 females) were collected from Şavşat Avcala plateau, Artvin province (Figure 1), during breeding season. Frogs were sexed following the presence of nuptial pad in males prior to cut fingertips, and morphological measurements were taken. Alive frogs were anesthetized using 250 mg/L MS222. After processing, samples were released where they were captured. The wounds were disinfected using Bactine^®^ spray (Wellspring Pharmaceutical, New York, NY, USA) containing benzalkonium chloride.

**Figure 2:**
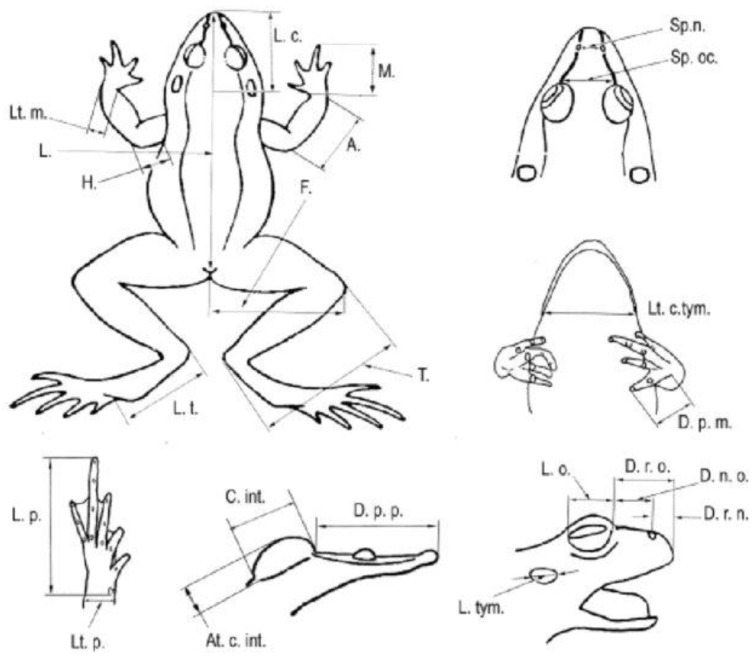
Schematic demonstration of the morphological measurements (this figure is taken from Peskov et al. [22]).

To explore the morphological patterns of the population based on linear measurements, 23 external characters (Figure 2) were measured using a digital caliper to the nearest 0.01 mm with the reference of Peskov et al. [22]: SVL (body length); L L. c.(head length); Lt. c. tym. (the head width at the tympanum level); D. r. n. (a distance from the nostrils to the end of the snout); Sp. n. (a distance between the nostrils); D. r. o. (a distance from the anterior margin of the eye to the end of the snout); D. n. o. (a distance from the nostril to the anterior margin of the eye); L. o. (eye slit length); L. tym. (tympanum length); Sp. oc. (a distance between the anterior margins of the eyes); A (forearm length); H (upper arm length); M (front foot length); D. p. m. (a length of the first digit of the forelimb); Lt. m. (wrist width); F (thigh length); T (shank length); L. t (tarsus length); L. p. (hind foot (pes) length); Lt. p. (hind foot width); D. p. p. (a length of the first toe of the hind limb); C. int. (a length of interior tuberculum calcanei); and At. c.int. (a width of interior tuberculum calcanei). Additionally, the specimens were weighed using the nearest 0.01 g electronic balance.

For age estimation using the skeletochronology method, the 4th toe of the hind limb of 54 frogs was cut off and preserved in 95% ethanol. Standard skeletochronology procedure was applied following Castanet and Smirina [23]. Phalanges were cleaned of soft tissues and preserved in 70% ethanol. The samples were washed in tap water for one hour and then decalcified with 5% nitric acid for approximately 1.5 h. The 18 μm cross-sections were obtained from second phalanges using a freezing microtome and stained with Ehrlich’s hematoxylin around 15 min. Photos were taken under microscope at 200× and 400× magnifications. Age estimation was carried out by counting the lines of arrested growth (LAGs). The calculation was performed by two independent researchers (S. Gül and C. Dursun). To avoid probable errors of the age estimation due to medullary resorption, diaphysis sections were taken into consideration. In agreement with other authors [24,25,26] and taking into account the microclimatic parameters of the sampling site, we assumed that each LAG corresponds to an annual arrest of individual growth.

### 2.2. Statistical Analyses

Descriptive statistics were calculated and summarized for both sexes, separately. The normality assumption of each variable was checked using the Kolmogorov–Smirnov test. To compare age, weight, and SVL between sexes (*n* = 54), non-parametric Wilcoxon test was executed. Thereafter, Pearson’s product-moment correlation test was used to estimate the relationships among these variables. The growth curve models were constructed under the typical Von Bertalanffy’s equation modified by Beverton and Holt [27]: Lt = L∞{1 − exp [−k(t − t_0_)]}, where Lt is the expected or average length at the time (or age) t, L∞ is the asymptotic average length, k is the so-called Brody growth rate coefficient, and t_0_ is a modeling artifact that is said to represent the time or age when the average length was zero. To visualize the growth curve, hypothetical individuals were added to the dataset by the reference of Socha and Ogielska [28] under the presented parameters: SVL_0_ at metamorphosis is fixed to a mean of 16.23 for females and 16.55 for males and t_0_ (age at metamorphosis) is 0.3 year. The calculations were obtained from body size measurements of 36 female and 35 male frogs at metamorphosis. For weight models, the value is fixed to 0.4 g in both sexes at the same age of metamorphosis. To estimate growth parameters and run the analyses, *FSA* v0.8.32 [29], *FSAdata* 0.3.9 [30], *FSAsim* v0.6.9 [31], and *nlstools* v2.0 [32] packages were used following the guide “fishR Vignette” [33].

For the second part of analyses, a total of 22 individuals including equal samples for both sexes were handled using morphometric variables except SVL. Since the variables showed a normal distribution (*p* > 0.05), downstream analyses were conducted following parametric tests. To reveal size differences between sexes, Student *t*-test was utilized by comparing the mean differences. For this purpose, each variable was divided into SVL to standardize data and reduce the body size effect on other variables. Thereafter, the dataset was subjected to the PCA to represent discrimination of sexes in morphospace. Lastly, the sexual shape dimorphism associated with the measured characters was tested following the analytic framework referenced in Dursun et al. [34]. Accordingly, ANCOVA analysis was carried out for all variables with sex as a factor and PC1 scores obtained from PCA as a covariate. To determine the direction of sexual shape dimorphism, the post hoc tests were conducted under Bonferroni correction. Statistical analyses were run using *stats* package v4.2.1 [35]. The results were visualized in *ggplot2* v3.3.6 [36]. All analyses were performed using R Programming Language v4.1.2 [35].

## 3. Results

The descriptive statistics of measurements for both sexes were presented in Table 1.

**Figure 3:**
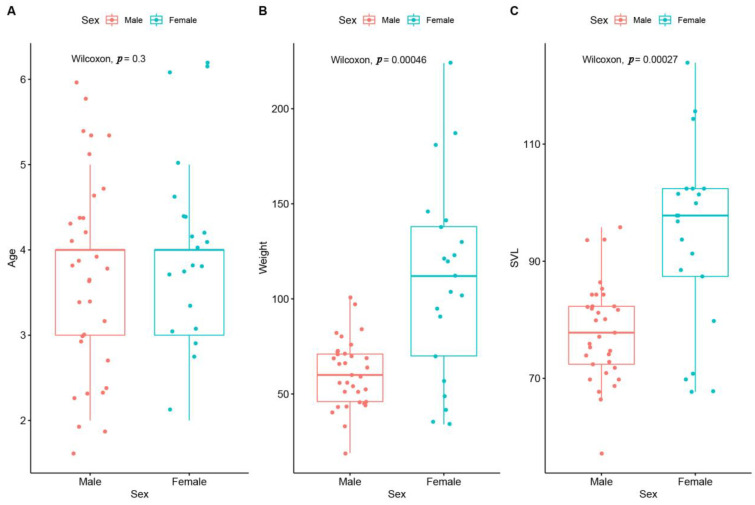
Differences between sexes are illustrated by boxplots. Points represent scattering of each individual (**A**: age; **B**: weight; and **C**: SVL).

According to Wilcoxon test results, significant differences were found between sexes in terms of SVL (W = 141; *p* < 0.05; female-biased) and weight (W = 775; *p* < 0.001; female-biased) but not in age (W = 290; *p* > 0.05). The results are visualized using boxplots in Figure 3.

**Figure 4:**
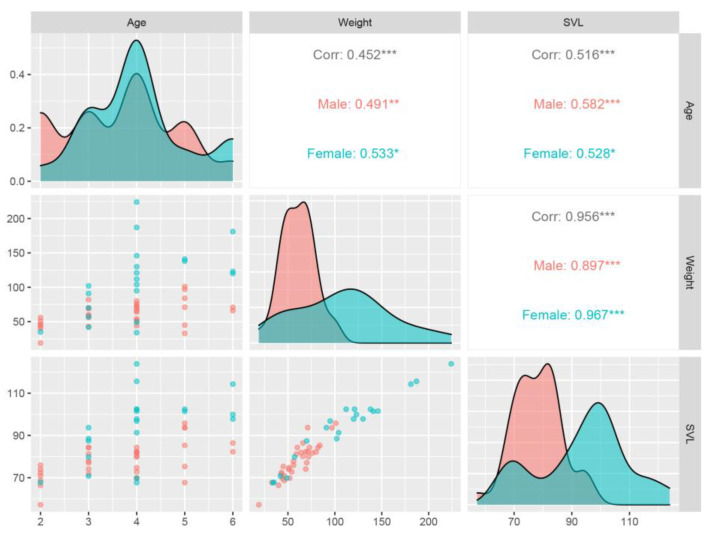
Pairs plot with correlation matrix. Corr values indicate the correlation coefficients (r). The significance level of correlation coefficients is represented with asterisk(s) (*, *p* < 0.05; **, *p* < 0.01; ***, *p* < 0.001).

Pearson’s product-moment test indicated that statistically significant positive correlations existed between the following variables (Figure 4). The constructed regression models also validated the linear relationship between age and SVL (males: F = 15.91; R^2^ = 0.31; *p* < 0.05; females: F = 7.35; R^2^ = 0.24; *p* < 0.05), age and weight (males: F = 9.82; R^2^ = 0.21; *p* < 0.05; females: F = 7.52; R^2^ = 0.24; *p* < 0.05), and weight and SVL (males: F = 127.30; R^2^ = 0.79; *p* < 0.05; females: F = 270.30; R^2^ = 0.93; *p* < 0.05).

**Figure 5:**
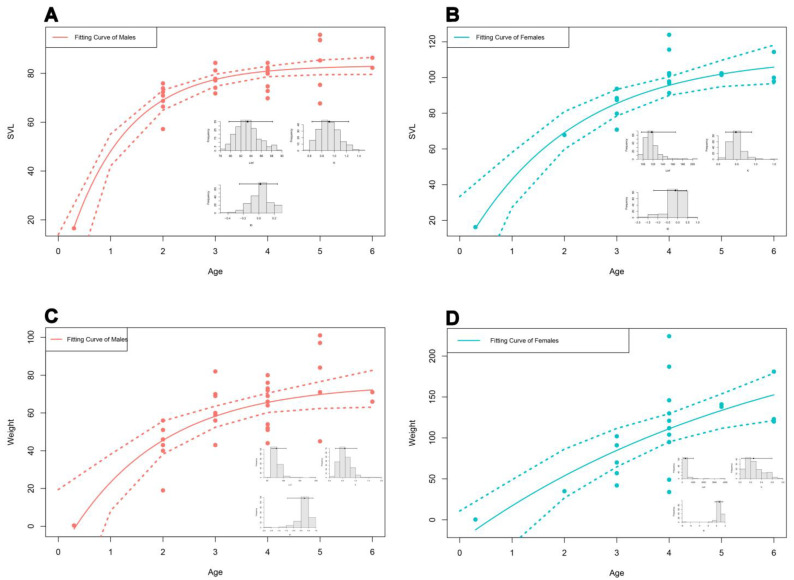
The constructed Von Bertalanffy’s growth curve models for age and SVL as well as age and weight (**A**: SVL model for males; **B**: SVL model for females; **C**: weight model for males; and **D**: weight model for females).

Growth curves under Von Bertalanffy’s model adequately fit the relationship between age, SVL, and weight. The curves indicated similar shapes in both sexes (Figure 5). The final models were found to be statistically significant for all parameters (*p* < 0.05). The growth parameters are presented in Table 2.

**Figure 6:**
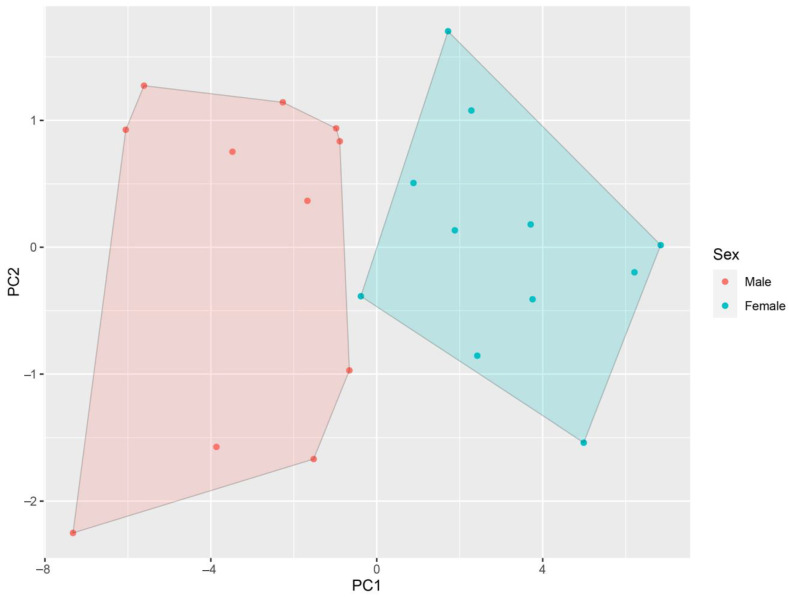
Scatterplot of SVL-independent PCA results based on 22 morphometric variables.

Student *t*-test results showed significant differences between terms of L. c. (*t* = 4.40; *p* < 0.001; male-biased), 0.61S. p. n. (*t* = 3.52; *p* < 0.001; male-biased), L. o. (*t* = 3.14; *p* < 0.001; male-biased), Lt. c. tym (*t* = 2.35; *p* < 0.05; male-biased), M (*t* = 4.33; *p* < 0.001; male-biased), F (*t* = 5.51; *p* < 0.001; male-biased), and At. c. int. (*t* = 2.17; *p* < 0.05; male-biased). Sexual shape dimorphism was observed only in M (F = 5.43; *p* < 0.05). Accordingly, males had larger forelimbs compared to females. SVL-independent PCA results showed that individuals from both sexes separated along PC1 (Figure 6). Three principal components were extracted, taking eigenvalues > 1 as a reference. The first principal component (PC1) explained 68.81% of the total variance. In total, 79.32% of variance was cumulatively explained by three components. PC loadings are noted in Table 3.

## 4. Discussion

In this study, the morphological population characteristics, the sexual variation in body size/shape, and age structure were investigated for a high-altitude-inhabitant population of *Pelophylax ridibundus*. The site at 2100 m is also the highest recorded site for the age structure studies of this species in Türkiye. Therefore, the results provided valuable information to increase the knowledge on the life history characteristics of the species.

As a basic phenomenon in anurans, it was found that females had larger mean values than males in all measured morphometric characters. The phenomenon known as sexual size dimorphism (SSD) is reported to be female-biased in 90% of the studied species [37,38]. A larger female body size is generally associated with the reproductive traits such as producing more offsprings and delayed sexual maturity to allocate more energy to growth, thus increasing reproductive success. Conversely, a smaller male body size is also advantageous to increase mobilization capacity and agility in breeding. Moreover, age differences between sexes, survival rates, and foraging completion can lead to SSD. In the genus *Pelophylax*, Disi and Amr [39] highlighted a significant difference in body size between sexes of *P. bedriagae* from Jordan (mean male SVL: 58.80 mm; mean female SVL: 61.70 mm). Gül et al. [20] revealed a significant size difference in the population of *P. ridibundus* from Lake Karagöl, Borçka (mean male SVL: 63.94 mm; mean female SVL: 72.96 mm). Fathinia et al. [40] assessed sexual size dimorphism in a *P. ridibundus* population distributed in Western Iran. They found significant differences in 12 morphometric characteristics, and females had larger mean values in each measurement (mean male SVL: 67.16 mm; mean female SVL: 78.36 mm), with well-discriminated scattering observed in PCA morphospace. They explained the observed difference via the fecundity selection hypothesis on female body size. Bamezar et al. [41] tested sexual dimorphism for *P. bedriagae* in Iran, and they found larger mean values for 13 different measurements (mean male SVL: 49.32 mm; mean female SVL: 64.04 mm). Additionally, genders were clearly discriminated in PCA morphospace, similar to findings in this study. The main reason of the observed difference was regarding a greater reproduction capacity of females and a higher mortality rate of males. Therefore, it can be thought that the SSD pattern observed for *P. ridibundus* is relevant for fecundity.

On the other hand, Amor et al. [42] assessed the morphological variation in *Pelophylax saharicus* in Northeastern Africa, and they found a lack of sexual dimorphism between sexes. Similarly, Pesarakloo et al. [43] investigated the taxonomic status of *P. bedriagae* populations in Iran, and they indicated that the lack of sexual differences between male and female water frogs. Svinin et al. [44] compared 11 different morphological indices between sexes of *P. ridibundus*, *P. lessonae*, and *P. esculentus* in Russia, and they found no sexual differences in any species. In amphibians, it is known that environmental gradients can affect body size variation. For instance, Johnson et al. [45] handled the body size evolution of ectotherms by analyzing 7270 amphibian species, and they reported that climate and elevation cause size differences. Olalla-Tárraga and Rodríguez [46] also said that thermoregulatory abilities among anurans facilitate reaching a larger body size based on the environmental energy. Bergmann’s rule also emphasizes that the population of a species living in colder environments tends to be larger than the population in warmer areas regarding energy conservation and thermoregulation [43,44], but it is completely applicable to all amphibians due to different life history traits and ecological interactions [47,48,49,50]. Therefore, different SSD patterns in the genus *Pelophylax* may be relevant to habitat conditions, species–specific life history traits, and thermoregulatory mechanisms.

Body weight differences between sexes of amphibians generally show a positive correlation with body size differences. For example, Seglie et al. [51] determined sexual dimorphism in a *Tylototriton verrucosus* population from the Himalayas, and they found that females were heavier (24.30 g) than males (13.30 g), as observed in SVL (95 mm and 81 mm, respectively). Yu et al. [52] examined body size and sexual size dimorphism in *Paa spinosa* from Ranidae family, and they calculated a high degree of correlation between body weight and SVL (89.20%). Otero et al. [53] also presented similar patterns in *Hypsiboas cordobae* between males (SVL: 48.01 mm; weight: 9.03 g) and females (SVL: 51.27 mm; weight: 12.30 g). Yılmaz et al. [17] evaluated the body size differences in *P. ridibundus* from the Yıldızlı stream population in Türkiye, and they presented the mean SVL and weight data of the sampled males (64.57 mm; 29.44 g; *n* = 38) and females (74.36 mm; 41.61 g; *n* = 11), in which females exhibited higher values. Based on previous findings, *P. ridibundus* followed the identical trend which is known as female-biased SSD in this study.

Pairwise comparison after reducing the body size effect on measurements indicated the presence of male-biased characters associated with the head dimensions (L. c., Sp. n., L. o., Lt. c. tym.) and limbs (M, F, At. c. int.). Accordingly, males have a larger and elongated head structure and bigger eyes than females. The variation observed in the head structure is reported for different anurans such as *Scutiger boulengeri* [54], *Bufo eichwaldi* [55], and *Charadrahyla sakbah* [56]. The main underlying reason for this variation was associated with the advantage to dislodge amplectant males on females because of the male–male competition shaped by similar evolutional and sexual pressures in distinct species.

For sexual shape dimorphism (SShD), only one of the twenty-three measured external characters showed significant difference between sexes. The character M (front foot length) had a larger value in males than that in females after reducing the size effect in data. The larger forelimbs provide more success in amplexus while grasping females [57,58], and it is a well-known trait of anurans [59,60,61]. Furthermore, Mao et al. [62] explored sexual dimorphism in the limb muscles in *Pelophylax nigromaculata*, and they said that the forelimb muscle structure was larger and heavier in males due to the adaptation of axillar amplexus. Petrović et al. [63] assessed SSD and SShD in terms of locomotion for nine amphibian species from Serbia to Montenegro, including *P. esculentus*, and they found that males have longer humerus and radioulna compared to their body size, which allow strongly holding females in amplexus. Therefore, the observed shape dimorphism in the forelimb of *P. ridibundus* is supported by the literature.

The age structure of the genus *Pelophylax* has become a subject of numerous studies in literature, and they are summarized in Table 4. The mean age ranged between 1.50 and 8 years for both sexes. The maximum age in the genus was calculated as 13 years, and the mean maximum age reported by these studies was 7.27 years. In this study, the maximum age was found to be 6 years, which is below average. For the mean ages, it was seen that the variation is independent of species.

In this study, the sampling region was placed at a higher altitude, and it is an important factor affecting the age structure and sexual dimorphism in amphibians. Phenotypic traits can demonstrate variation due to different adaptive strategies between sexes. Furthermore, variations can occur between populations along an altitudinal gradient. Baraquet et al. [91] investigated growth patterns and body size differences in *Boana cordobae* populations from 930 to 2130 m along an altitudinal gradient. They found that males at the higher altitudes were larger and older comparing to lower altitudes. Zhang et al. [92] explained altitudinal variation on the sexual dimorphism in *Nanorana parkeri* based on environmental conditions, and they observed that females lived longer and grew more slowly than males. Furthermore, the mean age was higher while growth rate was lower at high altitudes. The findings of this study showed similar patterns in terms of sexual dimorphism in the age structure of *P. ridibundus*. Females likely delayed reaching sexual maturity to invest energy for reproduction, thereby reach a larger body size. On the other hand, it was found that the mean age did not follow a trend of being higher compared to that in low-altitude populations as noted in Table 4. In anurans, individuals living at high altitudes tend to live longer because of slow growth patterns in metamorphosis and juvenile stages and the presence of fewer predators. However, it must be taken into consideration that the species has a broad distribution, and the age structure and body size variations were reported for various populations from the environments with different climatic conditions and selective pressures that cause phenotypic variability.

The relationship between SVL and age described by the von Bertalanffy growth curve model showed a significant and positive correlation in both sexes. The growth curve coefficient was higher in males than that in females. The peak growth of males was up to 3 years, and the growth rate sharply decreased between 3 and 6 years. For females, the peak was 4 years; however, the growth rate was gradually reduced by the age of 6 years. The same patterns were also observed in weight–age models. Considering k coefficient for *P. ridibundus*, *P. bedriagae*, and *P. caralitanus* in Table 4, it was seen that the range was between 0.16 and 0.76 (to view all values from different studies, please refer to Arısoy and Başkale [74]). In these studies, males had a higher growth coefficient in some populations as found in our study. It can be linked to the faster growth in males for reaching sexual maturity before females because females tend to invest their energy to produce more eggs by delaying their maturation. Furthermore, k values calculated in this study surpassed those reported in former studies. Thus, the altitude may play a role in determining the growth rate because of its effect on both intrinsic and extrinsic factors [93,94,95].

## 5. Conclusions

In conclusion, the study presented the age structure in a high-altitude-inhabitant population of *P. ridibundus* and provided useful data that can be utilized for comparison with other populations in further studies. Furthermore, sexual dimorphic traits were comprehensively explained to understand the effect of sexual pressures on body characteristics. It was also shown that the use of SVL alone is unreliable to estimate age, and it must be supported by skeletochronology.

## Figures and Tables

**Table 1:** Descriptive statistics of morphometric characters in this study (*n*: number of individuals; SE: standard error; Min.: minimum values; and Max.: maximum values). Measurements are presented in millimeter, age in year, and weight in gram.

	Males					Females				
Variable	*n*	Mean	SE	Min.	Max.	*n*	Mean	SE	Min.	Max.
SVL	33	78.05	1.46	57.20	95.80	21	93.95	3.43	67.70	123.90
Weight	33	60.87	3.10	19.00	101.00	21	109.61	11.19	34.00	224.00
Age	33	3.67	0.21	2.00	6.00	21	4.05	0.23	2.00	6.00
L. c.	11	21.35	0.58	18.20	23.90	11	23.96	1.07	19.10	29.20
Sp. n.	11	4.93	0.15	4.20	5.70	11	5.79	0.24	4.20	7.30
Sp. oc.	11	7.22	0.34	5.30	8.60	11	9.26	0.32	7.70	11.40
L. o.	11	8.82	0.32	7.30	10.80	11	10.72	0.27	9.70	12.50
L. tym.	11	5.30	0.15	4.40	6.30	11	7.3	0.42	5.10	10.30
D. r. o.	11	10.34	0.27	8.50	11.50	11	13.36	0.64	8.50	16.60
D. n. o.	11	4.30	0.15	3.30	5.20	11	5.56	0.19	4.80	6.70
D. r. n.	11	4.53	0.16	3.50	5.30	11	6.34	0.25	5.10	7.40
Lt. c. tym.	11	24.09	0.57	20.87	27.70	11	30.35	0.78	27.10	34.40
Lt. m.	11	6.88	0.27	5.30	8.300	11	8.55	0.56	6.10	11.50
M	11	17.08	0.60	13.4	19.80	11	19.98	0.75	15.60	24.00
A	11	14.09	0.61	10.6	17.70	11	19.08	0.94	14.40	23.90
H	11	10.79	0.44	9.30	13.50	11	12.99	0.73	9.00	18.10
F	11	37.94	1.21	30.5	43.40	11	48.21	1.24	42.40	54.60
T	11	32.94	1.10	27.10	40.10	11	42.55	1.12	38.40	48.90
L. t	11	19.73	0.87	15.20	22.92	11	25.26	0.78	20.50	29.90
At. c.int.	11	3.60	0.25	2.20	4.90	11	4.15	0.19	3.20	5.20
C. int.	11	8.47	0.28	7.10	9.80	11	12.14	0.61	9.20	15.50
D. p. p.	11	12.20	0.45	8.50	14.60	11	16.84	0.63	13.40	20.70
L. p.	11	37.22	1.25	29.90	42.60	11	46.75	1.99	30.90	54.10
Lt. p.	11	8.14	0.43	6.30	11.30	11	11.07	0.34	9.60	12.90
D. p. m.	11	11.08	0.61	8.50	14.60	11	14.41	0.59	11.10	17.70

**Table 2:** The constructed final model parameters for SVL and weight (CI: confidence interval; K: growth coefficient).

Estimated Parameters					
Age–SVL					
Sex	L∞	CI	K	CI	t_0_
Male	83.22	78.64–87.81	0.90	0.61–1.25	0.15
Female	112.21	97.96–157.78	0.47	0.20–0.85	0.09
Age–Weight					
Sex	W∞	CI	K	CI	t_0_
Male	75.45	63.55–108.61	0.55	0.22–1.05	0.29
Female	260.08	127.02–825.69	0.16	0.04–0.71	0.33

**Table 3:** Factor loadings of PCA and associated parameters.

Variables	PC1	PC2	PC3
L. c.	0.69	−0.23	−0.39
S. p. n.	0.77	0.18	0.16
Sp. o. c.	0.74	0.50	−0.23
L. o.	0.89	0.16	0.08
L. tym.	0.83	−0.05	−0.33
D. r. o.	0.75	−0.11	−0.40
D. n. o.	0.81	−0.18	−0.02
D. r. n.	0.84	−0.03	−0.17
Lt. c. tym	0.92	−0.07	−0.01
Lt. m.	0.70	0.07	−0.42
M	0.84	−0.16	0.04
A	0.90	−0.13	0.06
H	0.72	−0.37	0.25
F	0.97	0.05	0.08
T	0.96	0.04	0.11
L. t.	0.93	−0.13	0.22
At. c. int.	0.56	0.69	0.10
C. int.	0.89	−0.10	−0.06
D. p. p.	0.89	0.13	−0.02
L. p.	0.71	0.13	0.31
Lt. p.	0.90	−0.10	0.20
D. p. m.	0.79	−0.07	0.30
Eigenvalue	1.51	1.19	1.11
Variance (%)	68.81	5.45	5.06
Total variance (%)	68.81	74.26	79.32

**Table 4:** Age structure of the genus *Pelophylax* reported in the literature.

Species	Country	Sex	Mean Age	Max. Age	Study
*P. bedriagae*	Türkiye	Female	5.79	12	[64]
		Male	5.65	12	
*P. bedriagae*	Türkiye	Female	2.95	5	[65]
		Male	2.50	4	
*P. bedriagae*	Türkiye	Female	4.33	9	[66]
		Male	3.45	7	
*P. bedriagae*	Iran	Female	5.20	8	[67]
		Male	6.20	10	
*P. ridibundus*	Türkiye	Female	4.89	7	[20]
		Male	5.32	11	
*P. ridibundus*	Poland	Female	4.40	7	[28]
		Male	3.70	6	
*P. ridibundus*	Greece	Female	3.73	5	[18]
		Male	2.96	5	
*P. ridibundus*	Türkiye	Female	3.72	6	[17]
		Male	3.90	7	
*P. ridibundus*	Iran	Female	5.40	12	[68]
		Male	3.00	7	
*P. ridibundus*	Bulgaria	Female	−	5	[69]
		Male	−	5	
*P. ridibundus*	Croatia	Female	8.00	13	[70]
		Male			
*P. ridibundus*	Russia	Female	3.30	6	[21]
		Male	3.49	9	
*P. ridibundus*	Türkiye	Female	5.42	11	[71]
		Male	6.19	13	
*P. ridibundus*	Iran	Female	4.50	11	[72]
		Male	6.43	7	
*P. ridibundus*	Russia	Female	4.90	9	[73]
		Male	−	−	
*P. ridibundus*	Georgia	Female	4.03	7	[16]
		Male	2.78		
*P. caralitanus*	Türkiye	Female	5.23	10	[74]
		Male	4.59	9	
*P. caralitanus*	Türkiye	Female	6.01	10	[75]
		Male	5.01	9	
*P. caralitanus*	Türkiye	Female	5.66	10	[76]
		Male	4.90	9	
*P. lessonae*	Croatia	Female	4.80	8	[70]
		Male			
*P. porosus*	Japan	Female	2.00	4	[77]
		Male	1.50	4	
*P. porosus*	Japan	Female	1.66	4	[78]
		Male	1.55	3	
*P. pleuraden*	China	Female	2.81	4.5	[79]
		Male	2.56	3.5	
*P. terentievi*	Russia	Female	3.50	5	[80]
		Male	4.10	6	
*P. nigromaculatus*	China	Female	2.81	5	[81]
		Male	2.37	4	
*P. nigromaculatus*	South Korea	Female	−	−	[82]
		Male	4.44	8	
*P. nigromaculatus*	Japan	Female	4.09	6	[83]
		Male	3.34	6	
*P. perezi*	Spain	Female	−	6	[84]
		Male	−	4	
*P. perezi*	Spain	Female	2.01	6	[85]
		Male		5	
*P. epeiroticus*	Greece	Female	3.22	5	[86]
		Male	2.82	5	
*P. saharicus*	Algeria	Female	−	8	[87]
		Male	−	4	
*P. saharicus*	Morocco	Female	2.91	6	[88]
		Male	3.63	6	
*P. esculentus*	Sweden	Female	−	6	[89]
		Male	−	6	
*P. esculentus*	Romania	Female	6.70	10	[90]
		Male	5.00		
*P. esculentus*	Croatia	Female	5.10	10	[70]
		Male			

## Data Availability

Data available on request due to restrictions.
